# Association-heterogeneity mapping identifies an Asian-specific association of the *GTF2I* locus with rheumatoid arthritis

**DOI:** 10.1038/srep27563

**Published:** 2016-06-08

**Authors:** Kwangwoo Kim, So-Young Bang, Katsunori Ikari, Dae Hyun Yoo, Soo-Kyung Cho, Chan-Bum Choi, Yoon-Kyoung Sung, Tae-Hwan Kim, Jae-Bum Jun, Young Mo Kang, Chang-Hee Suh, Seung-Cheol Shim, Shin-Seok Lee, Jisoo Lee, Won Tae Chung, Seong-Kyu Kim, Jung-Yoon Choe, Shigeki Momohara, Atsuo Taniguchi, Hisashi Yamanaka, Swapan K. Nath, Hye-Soon Lee, Sang-Cheol Bae

**Affiliations:** 1 Department of Rheumatology, Hanyang University Hospital for Rheumatic Diseases, Seoul 133-792, Republic of Korea; 2 Institute of Rheumatology, Tokyo Women’s Medical University, Tokyo 162-0054, Japan; 3 Core Research for Evolutional Science and Technology (CREST), Japan Science and Technology Agency (JST), Tokyo 162-0054, Japan; 4 Division of Rheumatology, Department of Internal Medicine, Kyungpook National University School of Medicine, Daegu 700-721, Republic of Korea; 5 Department of Rheumatology, Ajou University School of Medicine, Suwon 443-380, Republic of Korea; 6 Division of Rheumatology, Daejeon Rheumatoid & Degenerative Arthritis Center, Chungnam National University Hospital, Daejeon 301-721, Republic of Korea; 7 Division of Rheumatology, Department of Internal Medicine, Chonnam National University Medical School and Hospital, Gwangju 501-757, Republic of Korea; 8 Division of Rheumatology, Department of Internal Medicine, Ewha Womans University School of Medicine, Seoul 158-710, Republic of Korea; 9 Division of Rheumatology, Department of internal medicine, Dong-A University, Busan 602-715, Republic of Korea; 10 Division of Rheumatology, Department of Internal Medicine, Arthritis & Autoimmunity Research Center, Catholic University of Daegu School of Medicine, Daegu 705-718, Republic of Korea; 11 Arthritis and Clinical Immunology Research Program, Oklahoma Medical Research Foundation, Oklahoma City, Oklahoma 73104, USA

## Abstract

Considerable sharing of disease alleles among populations is well-characterized in autoimmune disorders (e.g., rheumatoid arthritis), but there are some exceptional loci showing heterogenic association among populations. Here we investigated genetic variants with distinct effects on the development of rheumatoid arthritis in Asian and European populations. Ancestry-related association heterogeneity was examined using Cochran’s homogeneity tests for the disease association data from large Asian (n = 14,465; 9,299 discovery subjects and 5,166 validation subjects; 4 collections) and European (n = 45,790; 11 collections) rheumatoid arthritis case-control cohorts with Immunochip and genome-wide SNP array data. We identified significant heterogeneity between the two ancestries for the common variants in the *GTF2I* locus (*P*_Heterogeneity_ = 9.6 × 10^−9^ at rs73366469) and showed that this heterogeneity was due to an Asian-specific association effect (OR_Meta_ = 1.37 and *P*_Meta_ = 4.2 × 10^−13^ in Asians; OR_Meta_ = 1.00 and *P*_Meta_ = 1.00 in Europeans). Trans-ancestral comparison and bioinfomatics analysis revealed a plausibly causal or disease-variant-tagging SNP (rs117026326; in linkage disequilibrium with rs73366469), whose minor allele is common in Asians but rare in Europeans. In conclusion, we identified largest-ever effect on Asian rheumatoid arthritis across human non-HLA regions at *GTF2I* by heterogeneity mapping followed by replication studies, and pinpointed a possible causal variant.

Rheumatoid arthritis (RA) is an autoimmune disorder characterized by chronic joint inflammation and the presence of anti-citrullinated peptide antibody (ACPA)^1^. The genetic etiology of RA has been extensively investigated, especially in large-scale case-control studies that have identified about 100 RA-risk loci in European and Asian populations, including immune-related genes involved in cytokine signaling and the T-cell and B-cell pathways^2^. Additionally, genetic association studies using multiple ancestral cohorts have revealed a large overlap of the RA-risk alleles among different ancestries^2^,^3^,^4^,^5^, including between Korean and European populations^3^,^5^. Nevertheless, interesting exceptions (e.g., *PTPN22* and *PADI4*) showed heterogenic associations between European and Asian populations^6^. Several hypotheses may account for this heterogenic association. One of the simplest is that the presence of disease-causing allele is population-specific as the results of various genetic events such as natural selection, genetic drift, mutation or genetic migration^7^. Alternatively, population-specific differences in other genetic variants or environmental conditions may modulate the effects of disease-risk variants enormously through gene-gene and gene-environmental interaction with the disease-causing variant. In addition, genetic variants close to a causal allele may have a chance to show heterogeneity for their disease effects due to differences in linkage disequilibrium with the causal allele in different populations.

Here, we investigate the ancestry-related association heterogeneity using the association data from large Asian and European RA cohorts and newly identify an Asian-specific RA-risk variant in the *GTF2I* locus by means of a series of statistical and bioinformatics analyses.

## Results

### Identification of a heterogenic and Asian-specific disease association at the *GTF2I* locus

To investigate association heterogeneity due to ancestral differences, we revisited the previous data on trans-ancestral associations in high-density immune-loci genotyping-array (Immunochip) datasets (1 Korean and 7 European datasets) and genome-wide association study (GWAS) -derived imputed Immunochip datasets (1 Korean and 4 European datasets) that were generated from ACPA-positive RA cases and controls (n = 9,299 Koreans [2,234 cases and 7,065 controls] and n = 45,790 Europeans [10,288 cases and 35,502 controls])^3^. We compared the disease effect sizes of each genetic marker in the two ancestries using Cochran’s Q homogeneity tests based on inverse variance weights (see Patients and Methods for details).

By analyzing effect sizes for all 93,636 SNPs (λ = 1.03; Supplementary Material, Fig. S1), we found significant ancestry-related heterogeneity in the major histocompatibility complex (MHC) region, *PTPN22*, *GTF2I* and *UBASH3A*, exceeding the Immunochip-wide *P* threshold^8^ (*P* < 1.9 × 10^−6^), along with other possible heterogeneous loci including *PADI4*, *LCE1C* and *THRA* (Supplementary Material, Fig. S2). MHC, *PTPN22*, and *UBASH3A* have been previously reported to be associated with susceptibility to RA^2^.

Of these three loci, heterogeneity of the MHC region and *PTPN22* associations between Europeans and Asians was previously examined. In brief, the association between RA and MHC has been explained by the same HLA amino-acid haplotypes in Europeans and Asians. A residual effect within the MHC region was not detected in a conditional analysis adjusting the same amino-acid residues in the two populations^9^,^10^. Thus, it was likely that the observed heterogeneity in our unconditional analysis resulted from differences in linkage disequilibrium between particular SNPs and the causal amino-acid residues of HLA genes in the extended MHC region. For *PTPN22*, the RA risk allele of the nonsynonymous SNP rs2476601 was common in European populations but very rare in Asian populations^3^,^4^.

In *UBASH3A*, the strongest genetic heterogeneity between the populations was detected at the intronic SNP rs2839510, which is 8 kb from the most significant RA-associated SNP rs1893592 in both populations (*P*_*Heterogeneity*_ = 1.1 × 10^−6^; Supplementary Material Fig. S3). To understand the observed heterogeneity, the linkage disequilibrium between rs2839510 and rs1893592 was analyzed in European and Asian descendants. We found that in >99% of cases the minor allele *C* at rs2839510 was in linkage disequilibrium with the RA-protective allele *A* of rs1893592 in Asian individuals. In contrast, only ~1% were in linkage disequilibrium with the same allele in people of European descent (Supplementary Material, Table S1). This indicated that the distinct linkage disequilibrium patterns of the populations were responsible for the contrasting effects at rs2839510 in *UBASH3A*, as seen in the MHC region. The observed heterogeneity disappeared when the disease effects were conditioned on the RA-associated SNP rs1893592 (Supplementary Material, Fig. S4).

Genetic variations in the *GTF2I* locus have not been reported in previous association studies on RA. We found distinct effect sizes of rs73366469 in the two ancestries (*P*_*Heterogeneity*_ = 3.8 × 10^−7^). A significant association effect was seen in the Korean population (OR = 1.42; 95% CI = 1.26–1.59) but no effect was seen in European populations (OR = 1.00; 95% CI = 0.94–1.07). This heterogeneity resulted in insignificant association in a trans-ancestral meta-analysis (*P*_*Meta*_ = 4.1 × 10^−3^), which led the previous genetic study^3^ to consider the genotype calls and effect estimate of rs73366469 in Koreans to be unreliable and false positives. In fact, the significant heterogeneity and disease association in Koreans was detected for the single SNP (rs73366469) at this locus. Moreover, the SNP rs73366469 was genotyped only by Immunochip arrays (1 Korean and 7 European datasets) so that the association effect in Koreans was supported by a single dataset. Considering all these factors, we first genotyped rs73366469 in a subset of the Korean Immunochip individuals (n = 109) using a TaqMan SNP genotyping assay to validate genotype calls in the Korean Immunochip dataset. We confirmed that the genotyping by Immunochip was accurate, as the results of the different genotyping methods were 100% concordant (Supplementary Material, Fig. S5).

### Replicated association of the *GTF2I* SNP rs73366469 in Asian populations

**Figure 1:**
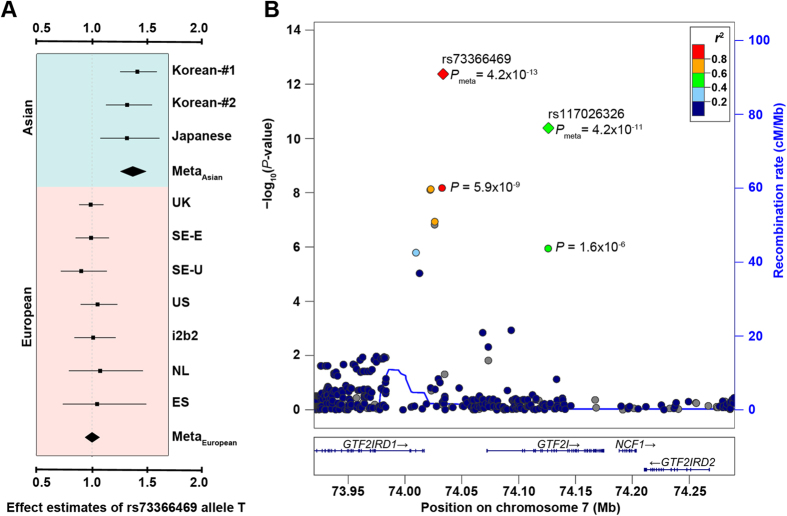
Association between *GTF2I* SNPs and rheumatoid arthritis in Asian populations. (**A**) Forest plot of the odds ratios of the minor allele *T* of rs73366469 associated with rheumatoid arthritis in each dataset. Korean-#1: Korean Immunochip collection; Korean-#2: Korean replication collection. (**B**) Regional association plot for association between rs73366469 and rheumatoid arthritis in Asian populations.

As only one Korean Immunochip dataset with information on rs73366469 was available, we conducted replication studies using two independent Asians cohorts to establish if the association of the *GTF2I* variant with RA was specific to Asian populations. We genotyped rs73366469 in an independent Korean cohort (1,263 ACPA-positive RA patients and 1,106 controls) and a Japanese cohort (2,029 ACPA-positive RA patients and 768 controls) using TaqMan assays. The call rates were ≥98% and the genotype results were under Hardy-Weinberg equilibrium (HWE; *P* ≥ 0.40 in controls; *P* ≥ 0.93 in cases). The association of rs73366469 was replicated in both cohorts as well as by a meta-analysis of these results along with original Korean Immunochip results (*P*_*Meta*_ = 4.2 × 10^−13^ and OR_Meta_ = 1.37; Fig. 1 and Supplementary Material, Table S2).

After combining all the data for rs73366469, the statistical significance of heterogeneity between Asians and Europeans was *P*_*Heterogeneity*_ = 9.6 × 10^−9^. In contrast, the SNP effects on susceptibility to RA were highly homogeneous among the Asian cohorts (*P*_*Heterogeneity*_  = 0.73, *I*^2^ = 0.0%) as were the disease effects of rs73366469 among the 7 European Immunochip datasets (*P*_*Heterogeneity*_ = 0.96, *I*^2^ = 0.0%; Fig. 1A; Supplementary Material, Tables S2 and S3).

### Identification of a potentially causal allele in the *GTF2I* locus conferring risk for RA in Asians

We imputed variations around the *GTF2I*-*GTF2IRD1* locus in the Korean Immunochip dataset to better localize the disease association signal. There were 8 SNPs with the Immunochip-wide *P* threshold of 1.9 × 10^−6^ or lower (Supplementary Material, Table S4), in contrast to none in the European Immunochip datasets (*P *> 0.001; data not shown). The most significant association was mapped to rs73366469 and three correlated proxy SNPs where significant heterogeneity was detected. A conditional analysis adjusting for rs73366469 found no evidence of additional secondary effects on susceptibility to RA (*P *> 0.001). One of these associated SNPs, rs117026326 (*P* = 1.1 × 10^−6^), was significantly associated with systemic lupus erythematosus in multiple Asians including Koreans^11^ and with primary Sjögren’s syndrome in Han Chinese^12^. All the associated SNPs spanned a >116-kb region harboring elements of *GTF2IRD1* and *GTF2I* (Fig. 1B).

Why does the heterogeneity occur at this particular locus? To address this question, we analyzed the minor allele frequencies and linkage disequilibria of the 8 Asian-specific RA-risk alleles in people of Asian and European descent. Although the SNPs were in higher linkage disequilibrium in Asians than in Europeans, each of the SNPs was in similar linkage disequilibrium pattern with the other SNPs in both Asians and Europeans. But there was an exception for rs117026326 among them. The average *r*^2^ between rs117026326 and the other SNPs was 0.44 in Asians but 0.04 in Europeans (Supplementary Material, Table S5). In addition, the minor allele of rs117026326 was rare in people of European descent, in contrast to those of Asian descent (minor allele frequency = 11.2% in Asians and 0.67% in Europeans; Supplementary Material, Table S5 and Fig. S6). It was thus tempting to suppose that the minor allele of rs117026326 was the source of the association with RA risk, therefore explaining the Asian-specific association. The very low frequency observed in European descendants provides essentially no power (0%) to detect associations with Immunochip-wide significance threshold or stronger in a typical association analysis, given the sample sizes of the European Immunochip datasets (n = 25,932) and the effect size in Asians (OR = 1.38).

We confirmed the association of rs117026326 in the Korean and Japanese replication cohorts and in a meta-analysis of the Asian datasets (OR = 1.38, 95% CI = 1.26–1.52 and *P* = 4.2 × 10^−11^; Supplementary Material, Table S6). The genotyping call rates were ≥98% and the genotype results were under HWE (*P* ≥ 0.61 in controls; *P* ≥ 0.53 in cases). Its effect was comparable to that of the lead SNP rs73366469.

### Association between rs117026326 genotypes and *GTF2IRD2* expression level

We investigated a possible regulatory role of rs117026326 in the expression of *GTF2I* and neighboring genes (*GTF2IRD1*, *GTF2IRD2* and *NCF1*) as the SNP is located in a non-coding element and is known to influence 6 potential regulatory motifs in an allele-specific manner (Supplementary Table S7). According to the data from the Human Protein Atlas Project^13^, *GTF2I*, *GTF2IRD1* and *GTF2IRD2* (encoding transcription factors and regulators) are expressed in most normal tissues including immune cells, and *NCF1* (encoding neutrophil cytosolic factor 1) is present in the cytoplasm of a subset of leukocytes.

We obtained gene expression data (lymphoblastoid cell lines) and rs117026326 genotypes in Asian HapMap participants from Stranger *et al.*^14^ and the 1000 Genomes Project, respectively. The normalized expression level of transcription factor gene *GTF2IRD2* was significantly associated with rs117026326 (nominal *P* = 4.8 × 10^−3^; Bonferroni-corrected *P* = 0.029). But there was no evidence that expression of the other genes was affected by rs117026326 (Supplementary Material, Fig. S7). Regulatory effect of rs117026326 on the expression of *GTF2IRD2* and other neighboring genes needs to be validated in more powerful expression quantitative trait loci (eQTL) study using various cell types and a larger sample size; in our *in-silico* eQTL analysis had no alternative but to analyze small number of Asian individuals who have been analyzed for both gene expression in lymphoblastoid cell lines and rs117026326.

## Discussion

This study investigated differences in the effects of immune-loci SNPs on RA in Asian and European populations. In our analysis, we identified four such loci showing significant heterogeneity for disease effects in the two populations. The heterogeneity involved (i) population-specific allelic heterogeneity of the causal SNPs in *GTF2I* and *PTPN22* and (ii) population-specific linkage disequilibrium of the lead RA SNP and other common SNPs in *UBASH3A* and the MHC region.

We demonstrated that the association of the *GTF2I* locus was restricted to Asian populations by mapping the heterogeneity and validating the association in Asian populations. *GTF2I* encodes the transcription factor TFII-I that binds to initiator and E-box elements in promoters^15^. TFII-I is activated by signal-induced phosphorylation and functions in pathways including immune signaling in activated B- and T-cells^15^. *GTF2IRD1* and *GTF2IRD2*, which are upstream and downstream of *GTF2I*, respectively, are also transcription factors or transcriptional regulators, but relatively little is known about the pathways in which they act. Hemizygous deletion of the *GTF2I* locus is associated with a rare neurodevelopmental disorder, Williams-Beuren syndrome^15^ but the copy number variation (CNV) of this locus among control individuals was reported not common (1%, n = 600)^16^. We observed HWE of the associated SNPs and a clear three-genotype-cluster plot with no evidence of common CNVs in our TaqMan results (e.g., Supplementary Material, Fig. S5). Similar findings were also observed in our recent GWAS for systemic lupus erythematosus in multiple Asian populations^11^ and a recent GWAS for primary Sjögren’s syndrome in Han Chinese populations^12^. Thus, this is unlikely that the RA-associated SNPs are genetically linked with the CNV, considering (i) HWE of the SNPs, (ii) the difference in the frequencies between SNPs and CNV, and (iii) no evidence of CNV in the TaqMan results, but there is a possibility of independent associations of the CNV with RA.

We suggested that the origin of the Asian-specific association in the *GTF2I* locus was located at rs117026326. The SNP rs117026326, a proxy of the initially detected SNP rs73366469, was significantly associated with RA and had the largest disease effect size in the locus. While the minor frequencies of all Asian-specific RA-associated SNPs did not differ much between Asian and European descendants, the RA-risk allele of rs117026326 seems to have been enriched in East Asian descendants over the population history, possibly due to geographical or demographic differences and genetic drift. Interestingly, rs117026326 was reported to be a lead SNP associated with systemic lupus erythematosus and primary Sjögren’s syndrome in Asian populations in recent studies^11^,^12^, but the association was not detected in extensive European populations in a previous Immunochip study or GWAS^17^,^18^, as was the cases for the association with RA in the present study. This implies that the risk effect of the *GTF2I* locus on RA may be widespread and Asian-specific in other immune disorders as well as in RA, systemic lupus erythematosus, and primary Sjögren’s syndrome.

The observed OR estimate of the minor allele at rs117026326 was 1.38, which was larger than those of all the previously associated SNPs in non-HLA loci in Asian populations^2^. Nevertheless, this effect had not been detected in other Asian RA association studies. That was simply because past GWAS arrays hardly if at all covered genetic variations at this locus. In fact, the GWAS data used in our study also lacked information on rs73366469 and its proxy SNPs, and imputing those SNPs from the GWAS data was unreliable and inaccurate. However, we were able to obtain evidence of an association between the *GTF2I* locus and RA because the Immunochip array provided genotype information for a single SNP (rs73366469) tagging rs117026326.

Similarly, recent studies for Asian populations using the immunochip or population-optimized array have identified the association of the same SNPs (rs73366469 and rs117026326) with systemic lupus erythematosus and primary Sjögren’s syndrome in Asians^11^,^12^. The effect estimates from the *GTF2I* variants were quite high (OR >2) and the significance levels were even stronger than HLA variants, although the *GTF2I* locus had not been detected before. Given the fraction of missing genetic heritability of RA^19^ and the existence of some genetic variants not tagged by current genome-wide SNP arrays, it is likely that many novel disease-associated loci are still hidden, for examples, in low-density genotyped loci and recombination hotspot loci.

By mapping the heterogeneity, we also found three loci (*PADI4*, *LCE1C* and *THRA*) that seemed to have distinct effect sizes in Asians and Europeans (*P*_*heterogeneity*_ < 5.0 × 10^−5^; Supplementary Material, Fig. S2). The association of exonic SNPs in *PADI4* (encoding citrullinating enzyme peptidylarginine deiminase 4) with RA was first discovered in the Japanese population in 2003 and validated in Korean and other Asian populations^20^,^21^. The association of the same SNPs was not replicated in European populations with a highly reliable significance level over the years although the SNPs were polymorphic in European populations. However, in 2012, the association was observed at rs2240336 in Intron 9 of *PADI4*, instead of at the exonic SNPs, in a large European study using the Immunochip array that provided high-density SNP data for *PADI4*. This difference in association results between Asian and Europeans was later explained in two trans-ancestral association mapping studies using Asian and European populations, in both of which high-density SNP data identified the most significant association at the intronic rs2301888^2,3^. Therefore, the previously observed heterogeneity in *PADI4* may have resulted from different patterns of linkage disequilibrium of the lead SNP and other common SNPs in two populations. Alternatively, population-specific secondary effects within the loci may cause the heterogeneity.

In summary, this study reinforces the importance of investigating population-specific disease-risk variants as well as shared risk variants for complex diseases such as RA. We identified the largest effect on RA across human non-HLA regions at *GTF2I* by heterogeneity mapping followed by replication studies, and found a potentially functional causal or disease-variant-tagging SNP within the *GTF2I* locus.

## Patients And Methods

### Study subjects and ethics

We analyzed individual-level genotype data on Asian participants, and the European association results were obtained from the summary statistics of 11 European case-control datasets consisting of 7 Immunochip and 4 GWAS datasets in a previous study^3^. The European collections have been previously described^3^,^22^. Briefly, the number of the European participants was 45,790, including 10,288 ACPA-positive RA cases and 35,502 controls. The participants were mainly from the United Kingdom and United States, and also included some Spanish, Dutch, Swedish and Canadian individuals. The Immunochip and GWAS array data were filtered based on standard quality control criteria for call rates per SNP or individual, minor allele frequency, HWE, imputation quality, population stratification, and/or cryptic relatedness, as previously described^3^.

The Asian subjects consisted of 4 case-control collections from 1 Immunochip array, one GWAS array and two replication cohorts generated from Korean and Japanese populations. Details of the participants and the quality control procedures employed in obtaining the immunochip and GWAS array data have been described^3^. Briefly, the participants were 9,299 ethnically homogenous Koreans (4,689 from Immunochip data; 4,610 from GWAS data), including 2,234 ACPA-positive RA cases and 7,065 controls. The Immunochip and GWAS array data were filtered based on standard quality control criteria for call rates per SNP or individual, minor allele frequency, HWE, imputation quality, population stratification, and/or cryptic relatedness, as previously described^3^. For the replication study on the association between RA and *GTF2I* SNPs, 5,166 participants were recruited from Hanyang University Hospital for Rheumatic Diseases (Seoul, South Korea; 1,263 ACPA-positive RA patients and 1,106 controls) and Tokyo Women’s Medical University (Tokyo, Japan; 2,029 ACPA-positive RA patients and 768 controls). Patients with RA in the replication cohorts fulfilled the 1987 criteria of the American College of Rheumatology^23^. ACPA levels were measured using ImmuLisa CCP2 ELISA kits. Anti-cyclic citrullinated peptide (anti-CCP) >25 units/mL was considered positive for ACPA. This study was approved for testing Asian individual-level genotype data by the Institutional Review Boards of Hanyang University (HYG-12-006-2, HYI-15-031-2) and Tokyo Women’s Medical University (217C), and written informed consent was obtained from each participant. All procedures for genotyping were carried out in accordance with the approved guidelines.

### Testing heterogeneity

Heterogeneity of disease effect sizes between ancestries for a given SNP was measured by Cochran’s homogeneity statistic (Q) using the following equation:


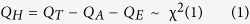


where 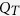 is the heterogeneity statistic from all the datasets (n ≤ 15; no more than 4 Asian collections and 11 European collections). 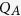 and 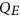 are the heterogeneity statistics from the Asian (n ≤ 4) and European datasets (n ≤ 11), respectively. The between-ancestry heterogeneity statistic (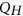) was calculated by subtracting the within-ancestry heterogeneity statistics (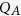 and 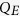) from the total heterogeneity index (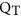); which follows a χ^2^ distribution with 1 degree of freedom under the null hypothesis. The genomic inflation factor in testing heterogeneity between ancestries was calculated using the most likely non-RA SNPs (n = 1,153) associated with writing and reading ability.

The Q statistic for a single SNP was calculated by Cochran’s homogeneity test based on inverse variance weights^24^ using the following equation:


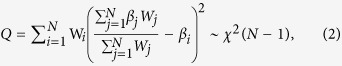


where 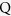 is the heterogeneity statistic for a SNP among 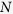 datasets. 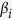 or 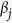
is the estimated effect on susceptibility to RA in the 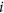 th or 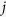 th dataset, and 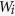 or 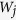 is the inverse of the variance of the effect (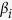 or 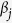) in the 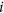 th or 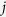 th dataset. The Q statistic follows an approximate χ^2^ distribution with 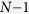 degree of freedom.

The significance threshold for the heterogeneity test was set as the Immunochip-wide significance level 1.9 × 10^−6^ defined by the Bonferroni correction for 26,146 independent SNPs (based on r^2^ < 0.05 in a sliding 1000-SNP window) in European populations of a previous Immunochip study on celiac disease^8^. We note that only 13,281 SNPs (based on r^2^ < 0.05 in a sliding 1000-SNP window) were independent in Korean Immunochip dataset but the portion of the independent SNPs in both the Korean and European populations was not examined because the individual-level genotype data for the European study cohorts was not available in this study.

The statistical power of Q test for two results (e.g., two population-specific effect sizes) is known to be relatively low^25^. The Q statistic for the difference in effect sizes between Asian and European populations are affected by the difference between two β of a tested allele and the β’s total variances. In addition, beta and variance are determined by sample size, allele frequency, relative risk, disease prevalence or others in two independent populations. Thus, the statistical power in our analysis is a very complicated outcome from multiple dynamic factors. Given the fixed total variance (=0.0030, the median of total variances in our discovery-stage analysis, that is manly affected by sample sizes) and the Immunochip-wide significance threshold, the heterogeneity showing ≥0.31 of beta difference between the populations was detected with ≥80% of statistical power. The statistical power was plotted in Supplementary Material Fig. S8, based on diverse beta differences and total variances.

### Imputing variations in the *GTF2I* locus

Genetic markers in a 1-Mb flanking region near the most significant SNP rs73366469 were imputed from the Korean Immunochip dataset by IMPUTE2^26^ based on the reference haplotype in the 1000 Genomes Project. Imputed data were filtered by Info ≥0.5 and MAF ≥1% before association analysis.

### Genotyping rs73366469 and rs117026326 using TaqMan assay

The two rheumatoid arthritis-associated SNPs rs73366469 and rs117026326 were genotyped for 109 Korean Immunochip samples and two replication cohorts (at SNP Genetics, Inc., Seoul, South Korea and Institute of Rheumatology or Tokyo Women’s Medical University, Tokyo, Japan) using the TaqMan SNP genotyping assay (Applied Biosystems, Foster City, CA, USA) according to the manufacturer’s instructions.

A premade primer/probe set was used for rs73366469 (Assay ID: C__97234117_10; Applied Biosystems) and a customized primer/probe set was used for genotyping rs117026326 (forward primer: 5′-GGT TAG TTT GCA TTT TCT ATA AAG TCT TAT GAA TGA AAT A-3′; reverse primer: 5′-GCT GTG GAT GAA TTT CAA AAC AAT CAT TT-3′; probe#1: 5′-CTC CCC GGC CCA TG-3′; probe#2: 5′-CTC CCC AGC CCA TG-3′). Polymerase chain reactions were performed using a 7900 Fast Real-Time thermal cycler or ViiA 7 Real-time PCR system (Applied Biosystems). The final reaction mixtures (5 μL) contained 5 ng genomic DNA, 2.5 μL 2X TaqMan Genotyping Master Mix, 0.125 μL 20X TaqMan SNP Genotyping Assay, and 2.375 μL double-distilled water. Thermal cycling conditions were 50 °C for 2 min and 95 °C for 10 min, followed by 40 cycles at 95 °C for 15 s and 60 °C for 60 s. Genotypes were determined using SDS 2.1 software (Applied Biosystems).

### Testing SNP associations with RA

Associations of SNPs with susceptibility to RA were examined by logistic regression including the top 10 principal component covariates obtained from the Korean Immunochip or GWAS data to adjust for potential population stratification, as previously described^3^. Principal component covariates were not available for the replication cohorts. Fixed-effect inverse-variance meta-analysis of the association results from each dataset was conducted by GWAMA^24^.

### Analyzing linkage disequilibrium and minor allele frequencies in Europeans and Asians

We obtained the variant calls for the Asian-specific rheumatoid arthritis associated SNPs (rs73366469, rs7800325, rs73366456, rs12667901, rs112502846, rs113066392, rs117026326, and rs2019004) from the Asian and European descendants who were analyzed in the 1000 Genomes Project. The Asians included 94 Japanese in Tokyo, Japan (JPT) and 91 Han Chinese in Bejing (CHB). Europeans were Utah residents with Northern and Western European ancestry (CEU), British in England and Scotland (GBR), and Iberian populations in Spain (IBS). All selected subpopulations were closely ethnically matched with the study participants. Linkage disequilibrium indices (*r*^2^) and minor allele frequencies were calculated from the variant call data.

### Testing the association between rs117026326 and gene expression

Among the Asian participants genotyped for rs117026326 in the 1000 Genomes Project, 85 from JPT and CHB were measured for gene expression using the Illumina Human WG-6 array in Stranger *et al.*^14^. (Note that rs117026326 was not genotyped in the International HapMap Projects so that eQTL results at rs117026326 are not available in Stranger *et al.*^14^ or other eQTL databases.) Raw gene expression data were normalized on a log scale using a quantile normalization method across replicates of a single individual, and a median normalization method across individuals. Expression levels of genes +/− 300 kb near rs117026326 (6 probes for 4 genes) were tested for association with dosage of the minor allele *T* at rs117026326 by linear regression.

## Additional Information

**How to cite this article**: Kim, K. *et al.* Association-heterogeneity mapping identifies an Asian-specific association of the *GTF2I* locus with rheumatoid arthritis. *Sci. Rep.* **6**, 27563; doi: 10.1038/srep27563 (2016).

## Supplementary Material

Supplementary Information
