# Supplementary material for: Association-heterogeneity mapping identifies an Asian-specific association of the GTF2I locus with rheumatoid arthritis
Source: Sci Rep. 2016 Jun 8;6:27563. doi: 10.1038/srep27563 (PMC4897688; doi:10.1038/srep27563)
Supplement: Supplementary Information [file srep27563-s1.pdf]

Supplementary Material

Association-heterogeneity mapping identifies an Asian-specific association of the *GTF2I* locus with rheumatoid arthritis

Kwangwoo Kim[1,†], So-Young Bang[1,†], Katsunori Ikari[2,3], Dae Hyun Yoo[1], Soo-Kyung Cho[1], Chan-Bum Choi[1], Yoon-Kyoung Sung[1], Tae-Hwan Kim[1], Jae-Bum Jun[1], Young Mo Kang[4], Chang-Hee Suh[5], Seung-Cheol Shim[6], Shin-Seok Lee[7], Jisoo Lee[8], Won Tae Chung[9], Seong-Kyu Kim[10], Jung-Yoon Choe[10], Shigeki Momohara[2], Atsuo Taniguchi[2], Hisashi Yamanaka[2], Swapan K Nath[11], Hye-Soon Lee[1] and Sang-Cheol Bae[1,*]

[1]Department of Rheumatology, Hanyang University Hospital for Rheumatic Diseases, Seoul 133-792, Republic of Korea; [2]Institute of Rheumatology, Tokyo Women's Medical University, Tokyo 162-0054, Japan; [3]Core Research for Evolutional Science and Technology (CREST), Japan Science and Technology Agency (JST), Tokyo 162-0054, Japan; [4]Division of Rheumatology, Department of Internal Medicine, Kyungpook National University School of Medicine, Daegu 700-721, Republic of Korea; [5]Department of Rheumatology, Ajou University School of Medicine, Suwon 443-380, Republic of Korea; [6]Division of Rheumatology, Daejeon Rheumatoid & Degenerative Arthritis Center, Chungnam National University Hospital, Daejeon 301-721, Republic of Korea; [7]Division of Rheumatology, Department of Internal Medicine, Chonnam National University Medical School and Hospital, Gwangju 501-757, Republic of Korea; [8]Division of Rheumatology, Department of Internal Medicine, Ewha Womans University School of Medicine, Seoul 158-710, Republic of Korea; [9]Division of Rheumatology, Department of internal medicine, Dong-A University, Busan 602-715, Republic of Korea; [10]Division of Rheumatology, Department of Internal Medicine, Arthritis & Autoimmunity Research Center, Catholic University of Daegu School of Medicine, Daegu 705-718, Republic of Korea; [11]Arthritis and Clinical Immunology Research Program, Oklahoma Medical Research Foundation, Oklahoma City, Oklahoma 73104, USA.

*To whom correspondence should be addressed at: Hanyang University Hospital for Rheumatic Diseases, Seoul 133-792, Republic of Korea. Tel: +82 222909237; Fax: 82 222988231; Email: scbae@hanyang.ac.kr

[†]These authors contributed equally to this work.

Table S1. Frequency of the rs2839510-rs1893592 haplotype in *UBASH3A**

Haplotype		Asians				Europeans		
rs2839510	rs1893592	KOR#1	KOR#2	JPT	CHB	GBR	CEU	IBS
C	*C*	0.00	0.00	0.00	0.01	0.10	0.06	0.04
A	*C*	0.25	0.25	0.23	0.23	0.20	0.25	0.20
C	*A*	0.25	0.25	0.37	0.23	0.00	0.00	0.00
A	*A*	0.50	0.50	0.41	0.53	0.69	0.69	0.76

*KOR#1: Korean Immunochip cohort; KOR#2: Korean GWAS cohort; JPT: Japanese in Tokyo, Japan; CHB: Han Chinese in Bejing; CEU: Utah residents with Northern and Western European ancestry; GBR: British in England and Scotland; IBS: Iberian populations in Spain.

All selected subpopulations in the 1000 Genomes Project were closely ethnically matched with the study participants. Haplotypes were extracted from Phase 3 long-range haplotypes constructed in the 1000 Genomes Project.

Table S2. Replicated association of rs73366469 with rheumatoid arthritis in Asian populations

Collection	MAF		OR (95% CI)	*P*
	Case	Control		
Korean replication set (n=2,369)	0.175	0.137	1.33 (1.13-1.57)	5.24E-04
Japanese replication set (n=2,797)	0.114	0.089	1.32 (1.08-1.62)	7.24E-03
Meta-analysis* (n=9,855)	-	-	1.37 (1.26-1.52)	4.15E-13

*Fixed-effect meta-analysis was performed for one discovery dataset (Korean Immunochip dataset) and two independent replication datasets (Korean replication dataset and Japanese replication dataset).

MAF: minor allele frequency; OR: odds ratio; CI: confidence interval.

Table S3. No association of rs73366469 with rheumatoid arthritis in European Immunochip cohorts

Collections from Rheumatoid Arthritis Consortium International for Immunochip (RACI)*	MAF		OR (95% CI)	P
	Case	Control		
UK (n=10,836)	0.093	0.094	0.99 (0.88-1.10)	0.82
SE-E (Swedish EIRA; n=3,702)	0.100	0.102	0.99 (0.85-1.16)	0.91
SE-U (Swedish Umea; n=1,487)	0.122	0.147	0.90 (0.71-1.14)	0.37
US (n=3,937)	0.096	0.100	1.05 (0.90-1.23)	0.54
i2b2 (US; n=2,840)	0.101	0.106	1.01 (0.84-1.22)	0.91
NL (Dutch; n=2,334)	0.091	0.092	1.08 (0.79-1.47)	0.65
ES (Spanish; n=796)	0.098	0.096	1.05 (0.73-1.50)	0.80
Meta-analysis** (n=45,790)	-	-	1.00 (0.94-1.07)	1.00

*All RA patients in European Immunochip cohorts are positive for anti-citrullinated peptide antibody (ACPA).

**Fixed-effect meta-analysis.

MAF: minor allele frequency; OR: odds ratio; CI: confidence interval.

Table S4. Association of SNPs in the *GTF2I* locus with rheumatoid arthritis in the Korean Immunochip dataset

		MAF					
SNP	Allele*	Case	Control	OR (95% CI)	*P*	Imputed/ Genotyped	INFO (Impute2)
rs73366469	*C<T*	0.178	0.131	1.42 (1.26-1.60)	5.86E-09	Genotyped	1.00
rs7800325	*C<T*	0.175	0.129	1.42 (1.26-1.60)	6.65E-09	Imputed	0.92
rs73366456	*G<A*	0.165	0.121	1.43 (1.27-1.62)	7.36E-09	Imputed	0.77
rs12667901	*T<G*	0.165	0.120	1.43 (1.27-1.62)	7.82E-09	Imputed	0.78
rs112502846	*G<A*	0.140	0.102	1.43 (1.25-1.63)	1.16E-07	Imputed	0.73
rs113066392	*A<AC*	0.140	0.102	1.42 (1.25-1.63)	1.49E-07	Imputed	0.73
rs117026326	*T<C*	0.109	0.078	1.44 (1.24-1.67)	1.13E-06	Imputed	0.58
rs2019004	*G<A*	0.266	0.222	1.29 (1.16-1.43)	1.61E-06	Imputed	0.53

* Minor allele < major allele.

MAF: minor allele frequency; OR: odds ratio; CI: confidence interval.

Table S5. Linkage disequilibrium (r^2) of Asian-specific rheumatoid arthritis-associated SNPs*

(a) In Asians

SNP #1	MAF	SNP #2							
		rs2019004	rs12667901	rs73366456	rs113066392	rs112502846	rs7800325	rs73366469	rs117026326
rs2019004	0.2512		0.32	0.32	0.30	0.30	0.29	0.28	0.14
rs12667901	0.1087	0.32		1.00	0.90	0.90	0.85	0.82	0.50
rs73366456	0.1087	0.32	1.00		0.90	0.90	0.85	0.82	0.50
rs113066392	0.1039	0.30	0.90	0.90		1.00	0.81	0.78	0.46
rs112502846	0.1039	0.30	0.90	0.90	1.00		0.81	0.78	0.46
rs7800325	0.1256	0.29	0.85	0.85	0.81	0.81		0.98	0.49
rs73366469	0.1232	0.28	0.82	0.82	0.78	0.78	0.98		0.50
rs117026326	0.1063	0.14	0.50	0.50	0.46	0.46	0.49	0.50	
Average r^2 with SNP #2		0.28	0.76	0.76	0.74	0.74	0.72	0.71	0.44

(b) In Europeans

SNP #1	MAF	SNP #2							
		rs2019004	rs12667901	rs73366456	rs113066392	rs112502846	rs7800325	rs73366469	rs117026326
rs2019004	0.2609		0.10	0.10	0.25	0.25	0.25	0.25	0.01
rs12667901	0.0387	0.10		1.00	0.32	0.32	0.39	0.39	0.09
rs73366456	0.0387	0.10	1.00		0.32	0.32	0.39	0.39	0.09
rs113066392	0.0842	0.25	0.32	0.32		1.00	0.90	0.90	0.00
rs112502846	0.0842	0.25	0.32	0.32	1.00		0.90	0.90	0.00
rs7800325	0.0926	0.25	0.39	0.39	0.90	0.90		1.00	0.03
rs73366469	0.0926	0.25	0.39	0.39	0.90	0.90	1.00		0.03
rs117026326	0.0067	0.01	0.09	0.09	0.00	0.00	0.03	0.03	
Average r^2 with SNP #2		0.17	0.38	0.38	0.53	0.53	0.55	0.55	0.04

* All value were derived from genotype data in the 1000 Genomes Project (Asians: CHB+JPT; Europeans: CEU+GBR+IBS).

MAF: minor allele frequency.

Table S6. Replicated association of rs117026326 with rheumatoid arthritis

Collection	MAF		OR (95% CI)	P
	Case	Control		
Korean replication dataset	0.164	0.123	1.40 (1.18-1.65)	7.34E-05
Japanese replication dataset	0.110	0.088	1.27 (1.03-1.55)	2.20E-02
Meta-analysis*	-	-	1.38 (1.26-1.52)	4.17E-11

*Fixed-effect meta-analysis was performed for 1 discovery dataset (Korean Immunochip dataset) and two independent replication datasets (Korean replication dataset + Japanese replication dataset).

MAF: minor allele frequency; OR: odds ratio; CI: confidence interval.

Table S7. Regulatory motif changes caused by rs117026326 alleles*

		PWM Score		Sequence	
				Ref	ATTTTCATGGGCCGGGGAGCAGTGTGGCTTCTTTCATTTCAA
PWM	Strand	Ref	Alt	Alt	ATTTTCATGGGCTGGGGAGCAGTGTGGCTTCTTTCATTTCAA
CCNT2_disc2	-	12.5	13		RGGGBHGGGG
CEBPB_disc2	-	1.5	13.4		YSATTGGCT
EBF_disc2	+	11.1	11.6		SYYNDGGVNVSDS
GR_disc6	+	11.1	0.2		SNVDGVVSDSNVDS
NRSF_disc5	+	11.4	8		SSVDGSNSSKS
Pax-4_5	-	11.3	11.8		GKRRKRDKDNDDKVNBDDBBDHWWWWWTTY

* Information is from HaploReg (*Nucleic Acids Res*. 2012 Jan;40: D930-4.). Red, SNP position.
PWM: position weight matrices; Ref: reference allele; Alt: alternative allele.

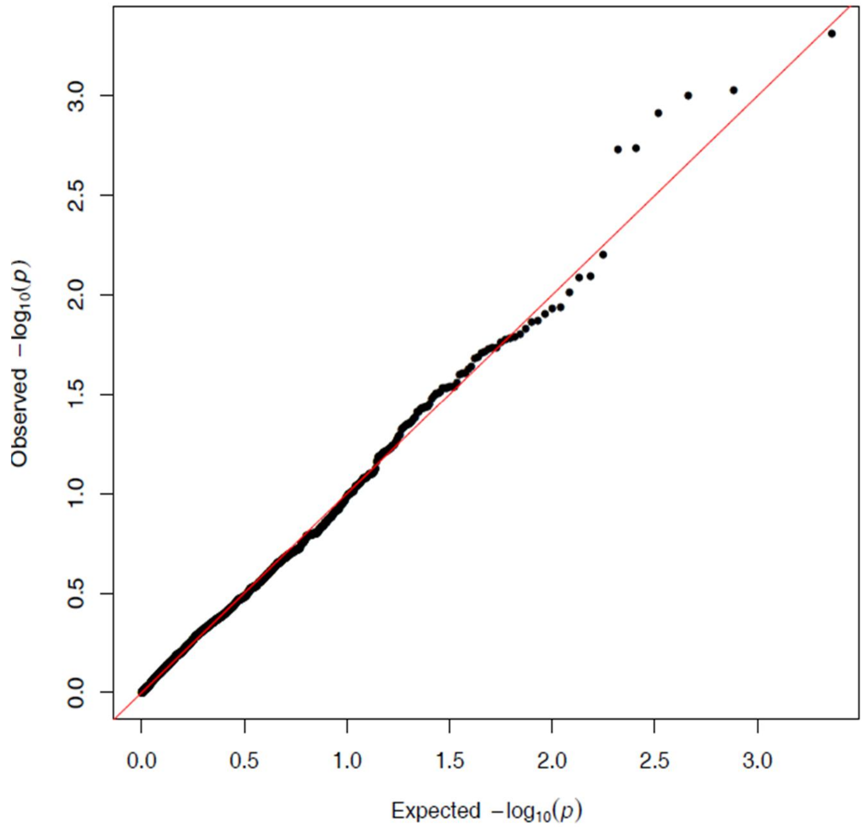

Figure S1. Quantile-quantile plot for heterogeneity tests. Observed probability was calculated from the most likely non-rheumatoid arthritis SNPs (n = 1,153) associated with writing and reading ability. Inflation factor λ was 1.03.

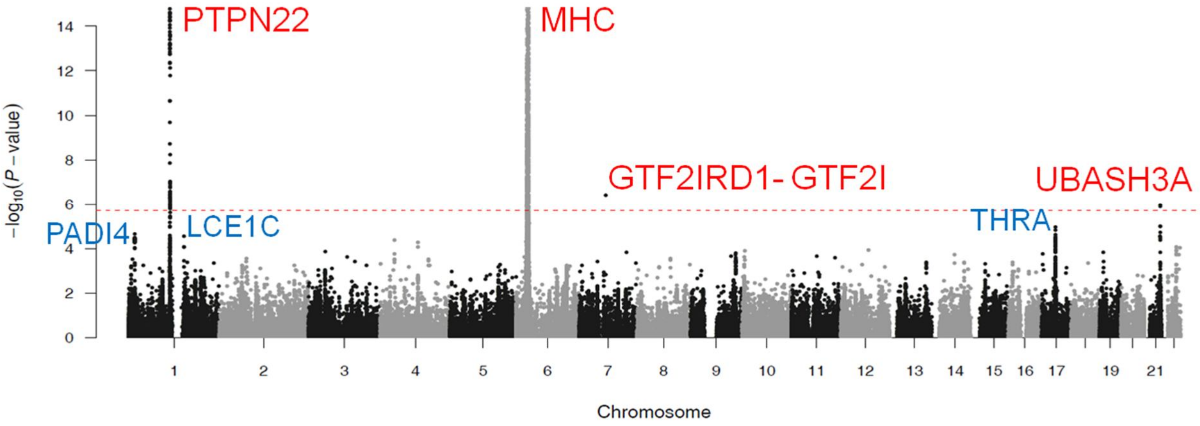

Figure S2. Manhattan plot for testing for heterogeneity of effect sizes between Korean and European datasets. Heterogeneity-associated loci passing the Immunochip-wide significance level (P<1.9×10^{-6}; dashed red line) are in red. Loci suggested to be potentially associated with heterogeneity (P<5×10^{-5}) are in blue.

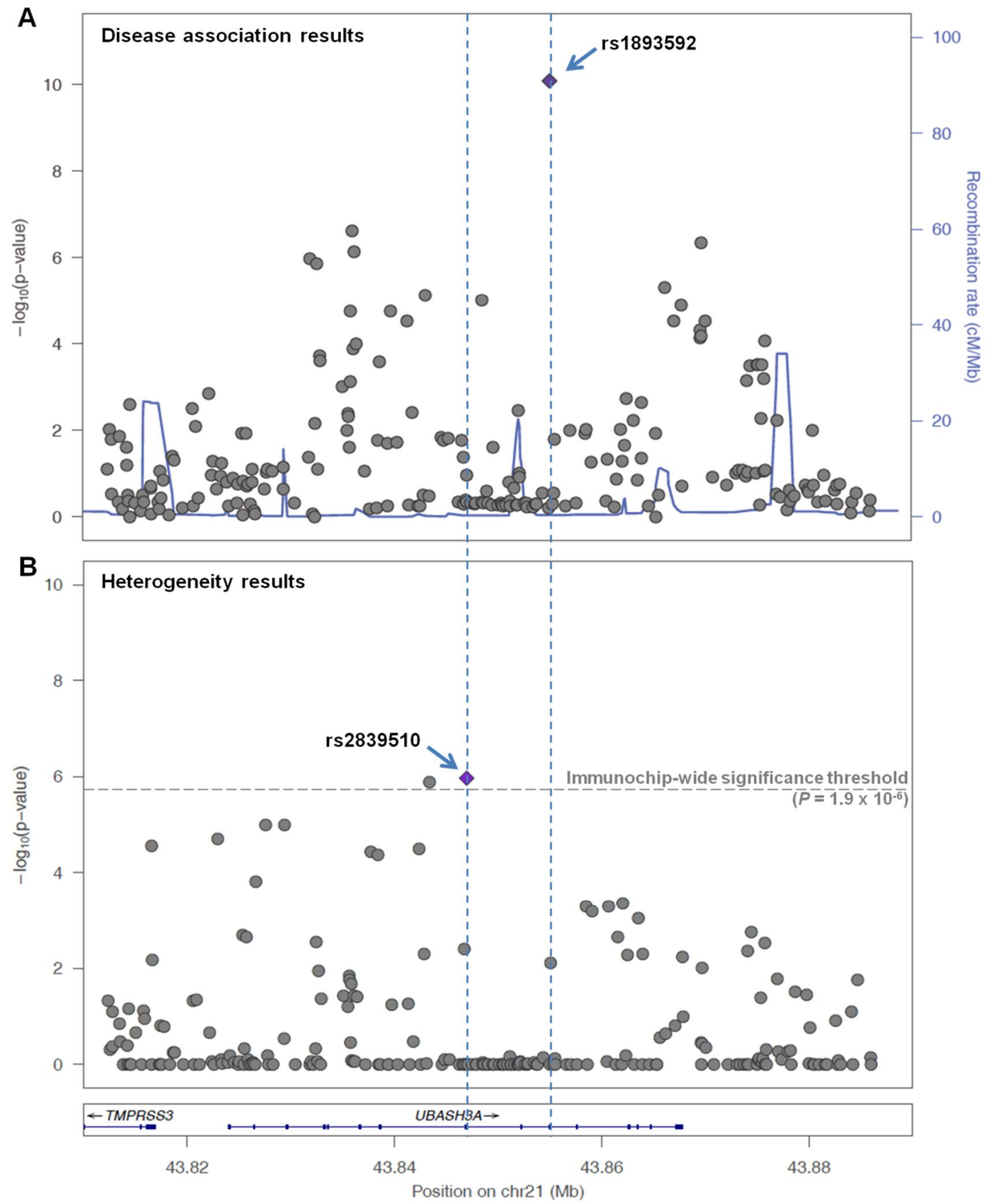

Figure S3. (A) Regional plot for association between *UBASH3A* and rheumatoid arthritis. Unconditional trans-ancestral meta-analysis identified the most significant association at rs1893592, as previously analyzed in Kim, *et al*. (*Ann Rheum Dis* 2015;74:e13). (B) Regional plot for heterogeneity of effect sizes between Koreans and Europeans. Significant heterogeneity was identified at rs2839510 (P=1.1×10^{-6}).

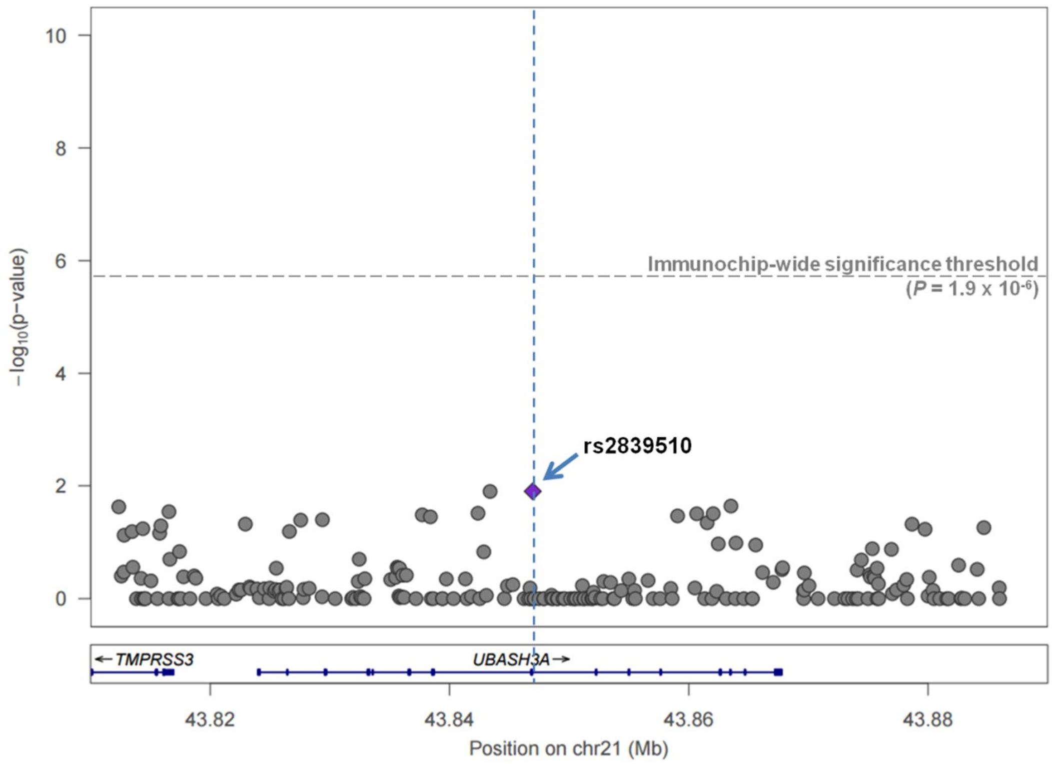

Figure S4. Regional plot for heterogeneity of effect sizes adjusted for rs1893592 in Koreans and Europeans. Effect sizes in each population were obtained by conditional logistic regression conditioning on the primary disease-associated SNP rs1893592. No SNPs, including rs2839510, in the *UBASH3A* locus showed heterogeneity.

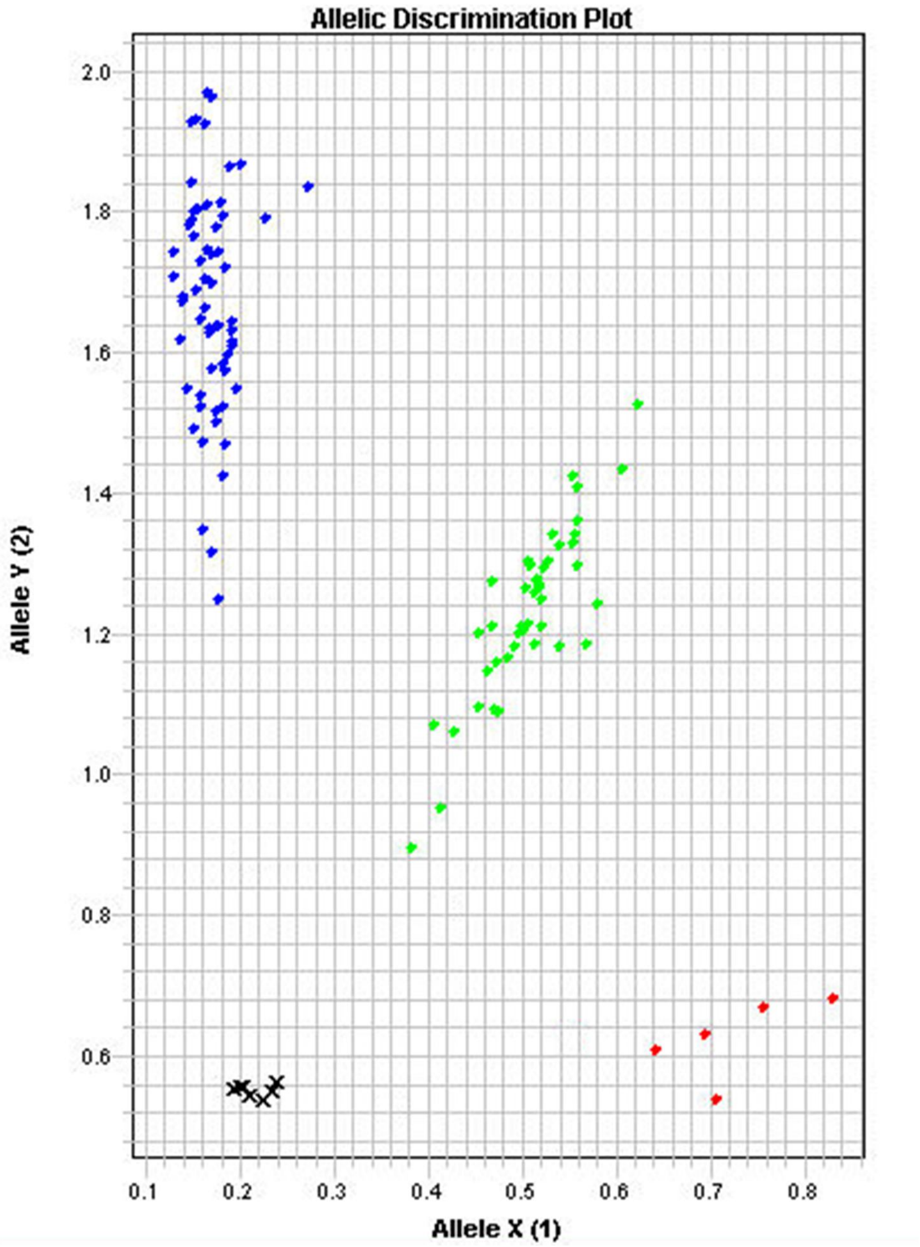

Figure S5. Cluster plot for rs73366469 in 109 Korean Immunochip samples by TaqMan assay. Blue indicates the homozygote TT (n = 62), green the heterozygote CT (n = 42) and red the homozygote CC (n = 5). Black indicates no call from negative controls. Genotyping calls in the TaqMan assay and Immunochip were 100% concordant.

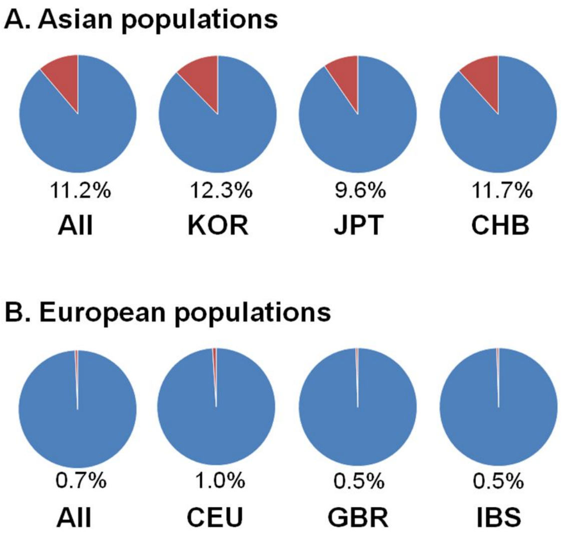

Figure S6. The frequency of the rs117026326 *T* allele in (A) Asian and (B) European populations. The frequency in Koreans (KOR) was calculated from the genotyping data for the Korean control participants used in the replication study. Subpopulations from the 1000 Genomes Project were closely matched to the study participants. JPT: Japanese in Tokyo, Japan; CHB: Han Chinese in Bejing; CEU: Utah residents with Northern and Western European ancestry; GBR: British in England and Scotland; IBS: Iberian populations in Spain.

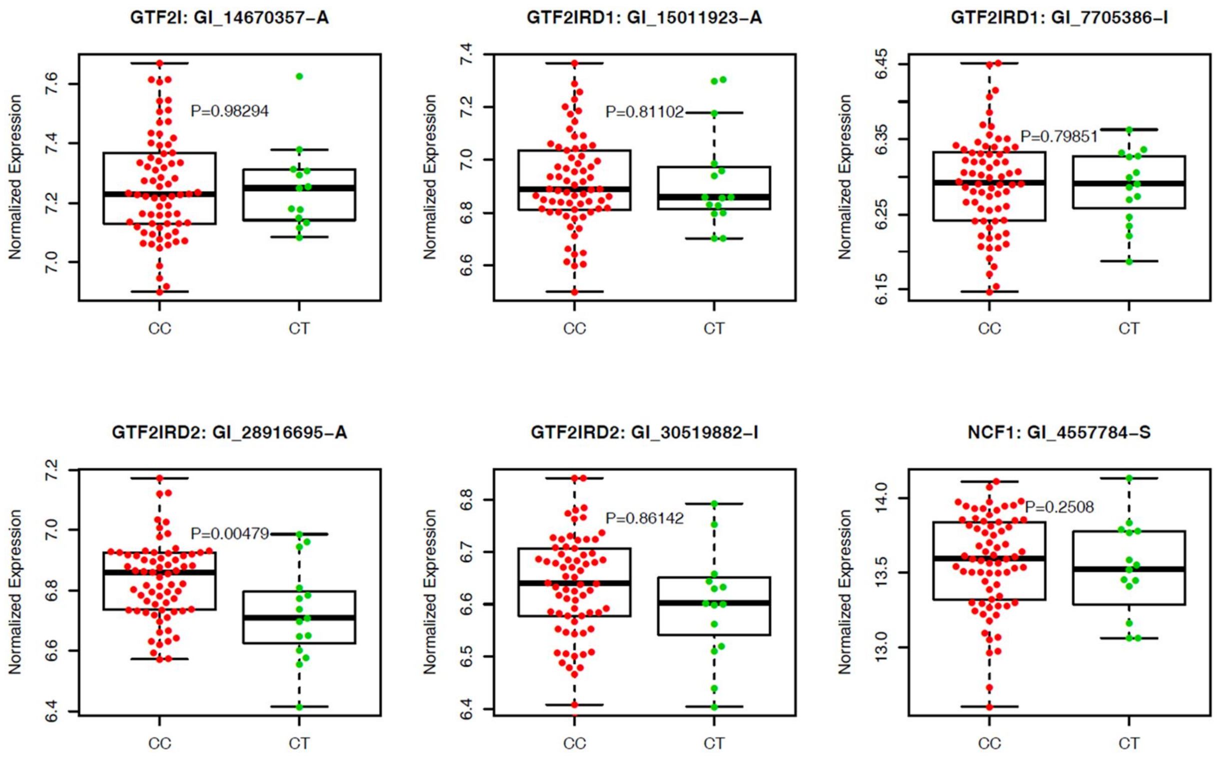

Figure S7. Association tests for the rs117026326 genotype and expression levels of *GTF2I*, *GTF2IRD*, *GTF2IRD2,* and *NCF1* in 85 Asian individuals (JPT and CHB). Expression levels of genes +/- 300 kb from rs117026326 (6 probes for 4 genes) were tested for association with the dosage of the minor allele *T* of rs117026326 using linear regression. Nominal P values are shown in the center of each plot.

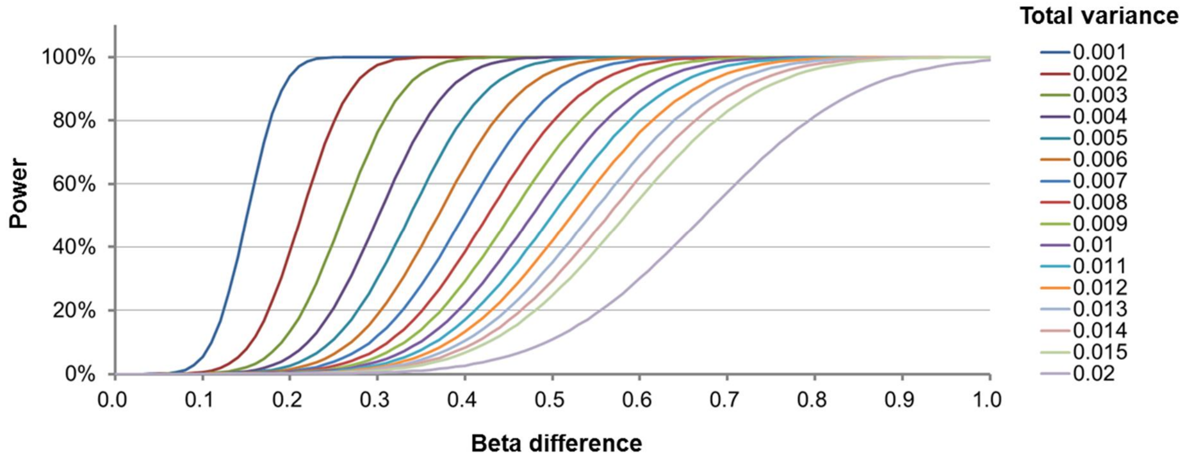

Figure S8. Statistical powers to detect heterogeneity at different beta difference and total variance. In our analysis, the Q statistic [= (β difference)^2/(Total variance)] follows a χ^2 distribution with 1 degree of freedom under the null hypothesis. With the median of total variances in our analysis (=0.003), more than 0.31 of beta difference between the populations can be detected with ≥80% of statistical power.
